# Systematic review on urinary continence rates after robot-assisted laparoscopic radical prostatectomy

**DOI:** 10.1007/s11845-023-03603-3

**Published:** 2024-01-10

**Authors:** Keith Geraghty, Kevin Keane, Niall Davis

**Affiliations:** 1 Department of Surgery, RCSI, Dublin, Ireland; 2 Urology Department, Beaumont Hospital, Dublin, Ireland

**Keywords:** Functional recovery, Prostate cancer, Radical prostatectomy, Robotic prostatectomy, Urinary incontinence

## Abstract

**Objective:**

The objective was to investigate the current evidence and discern urinary continence rates post robot-assisted laparoscopic radical prostatectomy (RALP).

**Methods:**

A systematic review of the literature was carried out, searching the Embase, Scopus and PubMed databases between 1 January 2000 and 1 May 2020. The search terms “*Robotic prostatectomy* AND *continence*” were employed. Articles were selected in accordance with the Preferred Reporting Items for Systematic Reviews and Meta-Analyses (PRISMA) guidelines. Statistical analysis was performed using the programme R; cumulative analysis of percentage of men continent was calculated.

**Results:**

A total of 3101 abstracts and 50 full text articles were assessed, with 22 publications included (*n* = 2813 patients). There were 21 randomised controlled trials and one partly randomised controlled trial with four publications comparing RALP to other prostate cancer treatments. Thirteen studies explored different RALP techniques, and five studies examined vesicourethral anastomosis (VUA). There were statistically significant improvements in early urinary continence rates in three studies analysing reconstructive techniques (83% vs 60%, *p* = 0.04; 26.5% vs 15.4%, *p* = 0.016; 77% vs 44.1%, p ≤ 0.001). Long-term continence rates were not significantly improved across all studies assessing reconstruction. One study comparing RALP vs laparoscopic radical prostatectomy (LRP) demonstrated a statistically significant improvement in continence at 3 months (80% vs 73.3%, *p* < 0.001); 6 months (83.3% vs 81.4%, *p* < 0.001); 12 months (95% vs 83.3%, *p* < 0.001) and 24 months (96.7% vs 85%, *p* < 0.001). Early continence was less favourable for RALP when compared to brachytherapy (BT) patients at 3 months (86% vs 98.7%, *p* < 0.05) and 6 months (90.5% vs 98.7%, *p* < 0.05).

**Conclusion:**

Early continence rates were improved across numerous techniques in RALP. These results were not translated into significantly improved long-term outcomes. Continence rates following RALP were favourable compared to LRP, similar to ORP and less favourable compared to BT. Our findings suggest that post-RALP continence can be further improved with alterations in robotic technique.

## Introduction

Prostate cancer is the second highest cause of cancer-related death in men [1]. Radical prostatectomy continues to have associated morbidity and an impact on patient quality of life [2, 3]. Considering this, there has been a relative paucity of experimental trials comparing different radical prostatectomy approaches, namely open retro-pubic radical prostatectomy (ORP), laparoscopic radical prostatectomy (LRP) and robot-assisted laparoscopic radical prostatectomy (RALP) [2]. Unlike other medical specialties, surgery presents unique challenges in producing quality RCTs for historical, commercial and technical reasons. Many surgical techniques were adopted as standard long before the concept of randomised trials was established. Once a procedure has been accepted by patient and doctor alike, it is difficult to get ethical approval to test it against a new and unknown technique. Financial challenges also exist; finely tuning a step in an operative procedure will not generate the same revenue for investors as a new cancer drug. Most importantly, the learning curve associated with a new procedure and variations in surgeon skill level introduces inherent bias that can be difficult to control for in surgical RCTs.

Long-term functional outcomes (i.e. urinary continence and potency) are reported as comparable across all three surgical approaches [3]. RALP is associated with less blood loss, longer operating times and higher cost compared to ORP [2–4]. However, since its introduction in 2000, RALP has become the most used and preferred surgical procedure for the treatment of prostate cancer [1]. While the literature is yet to prove RALP confers better oncological and functional outcomes than other approaches, surgeon preference for RALP seems due to several perceived technical advantages. These include greater instrument dexterity and 3D-magnified views allowing for nerve-sparing dissection, prostatic apex control and bladder neck preservation [4].

While different robotic approaches to prostatectomy have been developed, none have been conclusively shown to be more advantageous in terms of reducing urinary incontinence and erectile dysfunction rates, and the evidence for performing one approach over another remains vague [2, 3]. With significant heterogeneity in the available literature, and no accepted superior surgical approach for reducing post-operative urinary incontinence rates, this systematic review aims to add to the evidence base and guide treatment options for patients and urologists.

## Methods

### Search strategy

We conducted a systematic review in accordance with the Preferred Reporting Items for Systematic Reviews and Meta-analyses (PRISMA) statement and the Cochrane Handbook for Systematic Reviews of Interventions [5–7]. Embase, Scopus and PubMed databases were searched between 1 January 2000 and 1 May 2020 for relevant English language publications. The search strategy relied on the Patient Intervention Comparison Outcome (PICO) criteria [7]. The search terms “*robotic prostatectomy*” AND “*continence*” were applied.

**Fig. 1:**
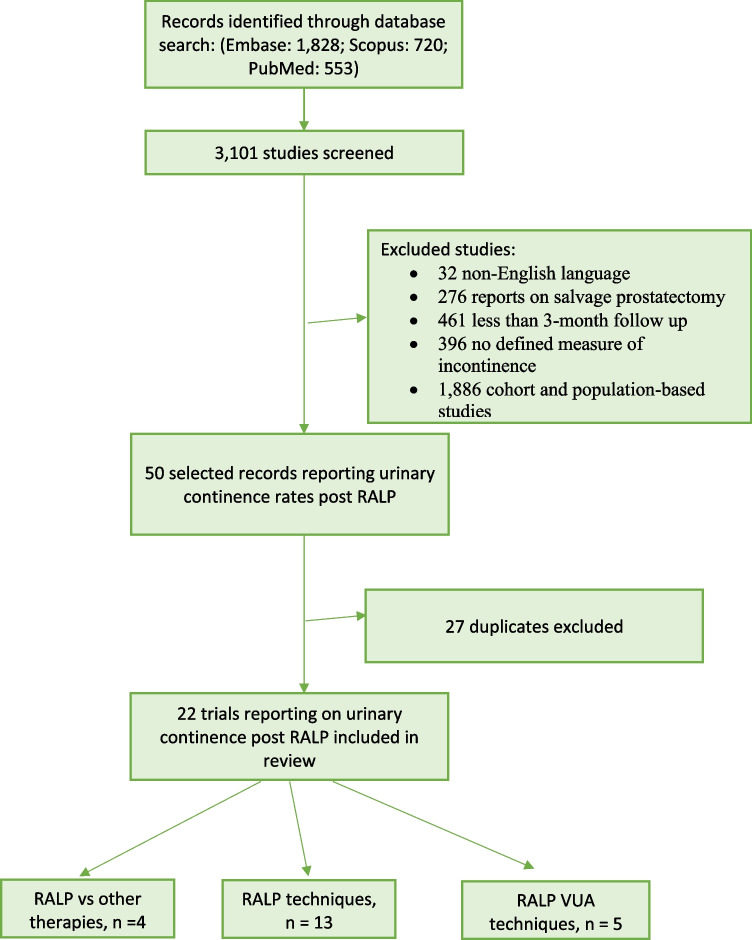
Flowchart of systematic review. RALP, Robot-assisted laparoscopic radical prostatectomy, VUA, vesicourethral anastomosis

Following de-duplication, two authors (K.G. and K.K.) independently screened the abstracts for eligibility as seen in Fig. 1. In the case of ambiguity related to the abstract, full texts were reviewed. Once the initial screening process was complete, the full-text articles were reviewed and scrutinised by the authors to confirm accordance with the inclusion criteria. The references of significant studies were then manually analysed to identify studies of interest.

### Types of study design included

Study designs were limited to prospective controlled, randomised and partially randomised trials. English language papers that reported urinary incontinence rates post RALP were included. For the purpose of this review, cohort studies, population-based studies, editorials, reviews, book chapters, commentaries, podium presentations and supplementary articles were excluded. Non-English papers, reports with less than 3-month follow up, studies reporting outcomes of salvage prostatectomy and studies failing to describe the measure of urinary continence were also excluded.

### Statistical analysis

Statistical analysis was performed using the “R” programme; version R for R 4.0.2 GUI 1.72 Catalina build (7847). Meta-analysis of percentage of men continent was employed, using the inverse variance method, DerSimonian-Laird estimator for tau^2^, Jackson method for confidence interval of tau^2^ and tau and the Freeman-Tukey double arcsine transformation. Forest plots of the meta-analysis were created for each subgroup analysed.

### Types of outcome measures included

The primary outcome measured was percentage of men continent following robotic prostatectomy, using any technique.

### Assessment of risks of bias

The risk of bias for RCTs was assessed in accordance with Cochrane guidance. This was facilitated by use of Version 2 of the Cochrane risk-of-bias tool for randomized controlled trials [8]. Only studies with low risk-of-bias were included.

## Results

### Description of the included studies

A total of 3101 abstracts were screened, and 50 full-text articles were assessed. Twenty-two were included following de-duplication. There were 21 randomised controlled trials, and one partly randomised controlled trial with 2813 eligible patients in total. Reasons for exclusion are listed in Fig. 1.

### Outcome data

Outcome data are summarised in Table 2. The study population, trial design, form of data collection, urinary continence rates at 3, 6, 12 and 24 months, definition of continence and presence or absence of validated questionnaire were recorded whenever available.

#### Studies comparing RALP to other treatments for prostate cancer

Four studies were included in this sub-analysis [9–12]. These studies compare RALP versus ORP, RALP versus LRP and, finally, RALP versus brachytherapy (BT). All studies showed 12-month continence rates > 90% with RALP.

In a 2018 study published by Coughlin et al*.*, a multitude of outcomes for RALP (*n* = 157) versus ORP (*n* = 151) were investigated. The authors described functional outcomes, reporting urinary continence rates (patients using zero pads per day) at the 6-, 12- and 24-month follow-up points of 84%, 90% and 91% post-RALP and 85%, 91% and 95% post-ORP [9]. They also reported the Expanded Prostate Cancer Index Composite (EPIC) questionnaire responses of patients, highlighting no significant difference between RALP and ORP (*p* < 0.0001) [9]. In a randomised controlled trial (RCT) by Porpiglia et al*.*, 5-year outcomes of RALP vs LRP were compared. They found continence rates of RALP group (*n* = 60) vs LRP group (*n* = 60) at 3 months (80% vs 73.3%, *p* < 0.001); 6 months (83.3% vs 81.4%, *p* < 0.001); 12 months (95% vs 83.3%, *p* < 0.001) and 24 months (96.7% vs 85%, *p* < 0.001) [10]. In 2011, Asimakapolous et al., compared RALP (*n* = 52) with LRP (*n* = 60) for the treatment of clinically localised prostate cancer. They noted continence rates of RALP vs LRP at 3 months (69% vs 63%, *p* = 0.51); at 6 months (88% vs 75%, *p* = 0.06) and at 12 months (94% vs 83%, *p* = 0.07) [11]. As seen in Table 1, Giberti et al*.* investigated RALP (*n* = 77) versus BT (*n* = 79) in a population of patients with low-risk prostate cancer. The study, defining continence as the use of zero pads per day, showed urinary continence rates for RALP vs BT patients at 3 months (86% vs 98.7%, *p* < 0.05); 6 months (90.5% vs 98.7%, *p* < 0.05); at 12 months (95.1% vs 98.7%) and at 24 months (95.1% vs 98.7%) [12].

**Table 1:** Urinary continence rates in studies comparing RALP to other treatments for prostate cancer

First author	Study design	Validated questionnaire	Continence definition	Cases, n	Group	Urinary continence rates, %				Level of evidence [5]
						3 m	6 m	12 m	24 m	
**Coughlin’18 **[9]	RCT	EPIC	0 pads/24 h	157	RALP	-	84	90	91	1b
				151	ORP		85	91	95	
**Porpiglia ‘16 **[10]	RCT	EPIC	≤ 1 pad/24 h	60	RALP	80	83.3	95	96.7	1b
				60	LRP	73.3	81.4	83.3	85	
**Asimakapolous '11 **[11]	RCT	None	0 pads/24 h	52	RALP	69	88	94	-	1b
				60	LRP	63	75	83		
**Giberti ‘17 **[12]	RCT	IPSS	0 pads/24 h	77	RALP	86	90.5	95.1	95.1	1b
				79	BT	98.7	98.7	98.7	98.7	

*RALP* robot-assisted laparoscopic prostatectomy, *ORP* Open retropubic radical prostatectomy, *LRP* laparoscopic radical prostatectomy, *BT* brachytherapy, RCT randomised controlled trial, *EPIC* Expanded Prostate Cancer Index Composite, *IPSS* International Prostate Symptom Score

#### Studies comparing RALP techniques

Fourteen RCTs assessed whether alterations in surgical technique had any impact on urinary continence rates following RALP.

One RCT examined the Retzius-sparing (RS-RALP) approach and showed favourable results for its impact on urinary continence. Menon et al*.* performed an RCT on 120 patients, comparing the RS-RALP technique (*n* = 60) and the standard/anterior RALP approach (*n* = 60). For the 3-, 6- and 12-month follow up, they found continence rates of 85% vs 93.3%; 93.3% vs 98.3% and 93.3% vs 98.3% for the anterior approach and RS-RALP approach respectively (*p* = 0.09) [13].

The utilization of intra-operative supportive slings was assessed in four studies [14–17]. Nguyen et al*.* studied the impact of a retropubic urethral sling on urinary continence post RALP. They reported urinary continence rates of 76% and 88% at 3- and 6-month intervals for the sling group (*n* = 95), while the control group (*n* = 100) had rates of 75% and 86% at the same periods (*p* = 0.87) [14]. In a similar study, Bahler et al*.*, examined a biologic bladder neck to a control group to determine the effect on post-RALP continence. They reported continence rates in sling vs control groups at 3 months (75% vs 74%, *p* = 0.84); 6 months (85% vs 86%, *p* = 0.79) and 12 months (95% vs 87%, *p* = 0.15) [15]. In a 2015 study, Cestari et al*.* evaluated the impact of a sub-urethral autologous sling vs control on 60 patients. The trial disclosed continence rates in the sling vs control groups at 3 months (83% vs 60%, *p* = 0.04); at 6 months (93% vs 73%, *p* = 0.03) and 12 months (97% vs 80%, *p* = 0.04) [16]. In a further study by Cestari et al*.* in 2017, the continence rates post RALP with two-branched sling (*n* = 60) vs six-branched sling (*n* = 60) were compared. The authors described continence rates of 98.3% in the six-branch sling and 95% in the two-branch sling at 12 months post operatively (*p* = 0.05) [17].

Four studies investigated the role techniques to restore anatomy following RALP have on post-operative urinary continence [18–21]. Atug et al*.* found improved urinary continence rates with the application of anterior and posterior anatomical reconstruction post-RALP [18]. They reported statistically significant improvements in very early continence for the anatomical reconstruction group at 1 week (*p* < 0.0001) and 1 month (*p* = 0.0002) [18]. The long-term continence rates in reconstruction (*n* = 125) vs control (*n* = 120) groups were at 3 months (80% vs 76%, *p* = 0.5176); 6 months (84% vs 80%, *p* = 0.5088) and 12 months (91% vs 88%, *p* = 0.5956) [18]. Hurtes et al. assessed the impact on urinary continence of anterior retropubic suspension and posterior reconstruction during RALP. According to the University of California Los Angeles Prostate Cancer Index (UCLA–PCI) definition of continence, the intervention group (*n* = 33) vs control group (*n* = 39) reported continence rates of 26.5% vs 15.4% (*p* = 0.016) at 3 months and 65.4% vs 57.9% (*p* = 0.609) [19]. In 2015, Jeong et al*.* studied a novel one-step posterior reconstruction method and its impact on post-RALP urinary continence. They discovered a significantly shorter time to social (≤ 1 pad/24 h) continence recovery in the intervention group (*n* = 50) (median 18 vs 30 days, *p* = 0.024) [20]. However, the overall continence rates were similar between groups (*p* = 0.89) [20]. Posterior rhabdosphincter reconstruction was applied by Sutherland et al. They concluded that posterior rhabdosphincter reconstruction offered no advantage for return of early continence post-RALP [21]. Rates reported were 63% and 81% for the intervention group (*n* = 47) and control group (*n* = 47) respectively, at 3 months post-procedure (*p* = 0.14) [21].

**Fig. 2:**
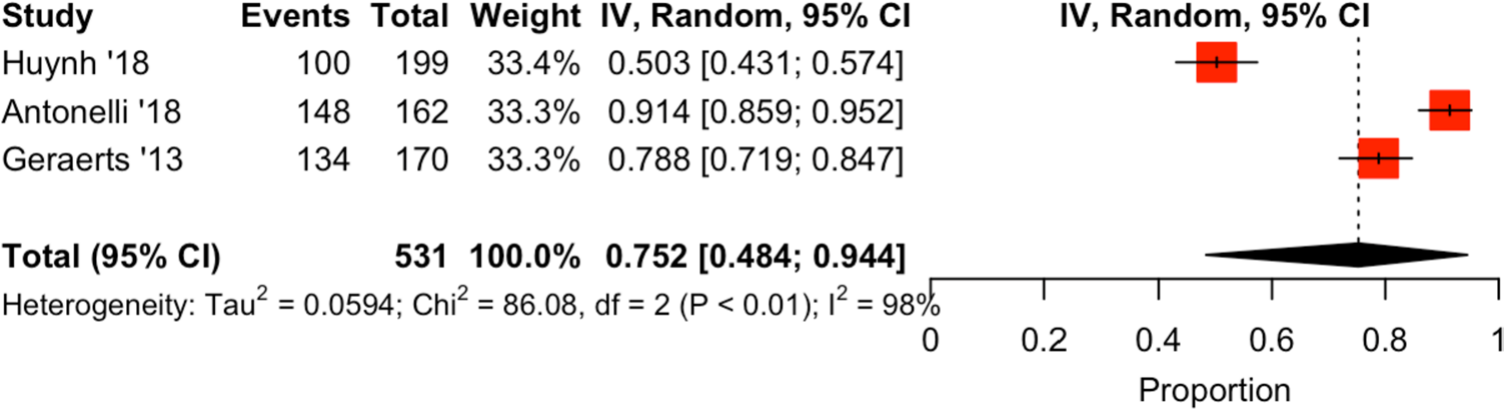
Cumulative analysis and forest plot—percentage of men continent post-RALP at 3 months in studies comparing different techniques in RALP

Four studies explored different RALP techniques which do not fall into any of the above categories [22–25]. Cumulative analysis can be seen in Fig. 2. Akand et al. compared two differing approaches to the RALP procedure: trans-peritoneal (T-RALP) and extra-peritoneal (E-RALP). They found increased urinary continence rates amongst the patients within the E-RALP approach group (*n* = 60) with patients reporting zero pads per day in 93.3% at 6 months [22]. The T-RALP group (*n* = 60) reported continence rates of 91.7% at the same period [22]. There was no statistical significance reported between the groups (*p* = 0.927) [22]. In a 2018 paper by Huynh et al*.*, the authors investigated the effect of regional hypothermia during RALP on urinary continence rates. In their RCT, they compared two parallel groups, finding no benefit in the 3-month continence rates in the regional hypothermia group reporting post-RALP continence of 50% and 59.6% in the regional hypothermia (*n* = 100) and control groups (*n* = 99) respectively (*p* = 0.1748) [23]. Antonelli et al*.*, explored standard versus delayed ligature of the dorsal vascular complex (DVC) during RALP in their RCT. The delayed-DVC suture group (*n* = 81) had rates of 91.4% and 98%, whereas the standard-DVC group (*n* = 81) had 95% and 97% at the 3- (*p* = 0.393) and 6-month (*p* = 0.752) follow up [24]. In a study by Geraerts et al*.*, the authors proposed that pre-operative pelvic floor muscle exercises (PFME) would increase early return to continence post-RALP. The results showed no significant difference between the continence in the groups at the 3- (*n* = 64, *p* = 0.80), 6- (*n* = 73, *p* = 0.264) or 12-month (*n* = 72, *p* = 0.083) stages [25].

#### Studies comparing anastomosis techniques

**Fig. 3:**
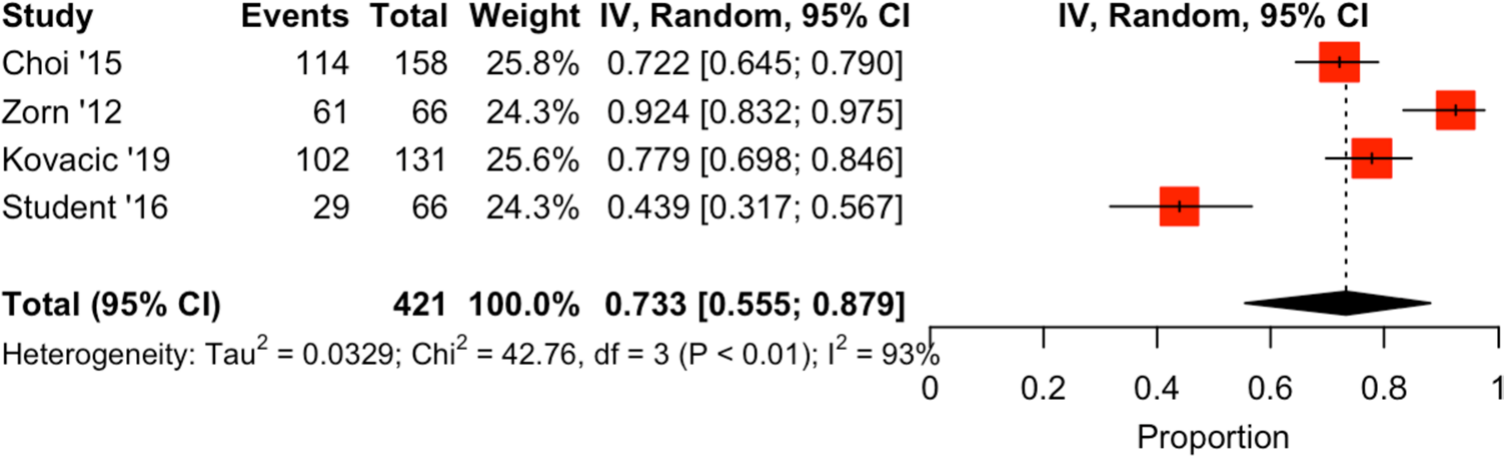
Cumulative analysis and forest plot—percentage of men continent post-RALP at 6 months in studies using different vesicourethral anastomosis techniques in RALP

Five trials, as seen in Fig. 3, investigated different techniques applied to the vesicourethral anastomosis (VUA). In a 2015 study, Choi et al*.* conducted an RCT comparing a bladder neck plication suture versus standard technique RALP. The intervention arm (*n* = 79) reported continence rates of 53.2% and 72.2% at 3 and 6 months post-RALP, whereas the control arm (*n* = 79) had rates of 59.5% and 74.7% (3 months *p* = 0.423, 6 months *p* = 0.719) [26]. The Advanced Reconstruction of Vesicourethral Support (ARVUS) technique was evaluated by Kovacik et al*.* in 2019. Their partially randomized, blinded trial investigated 1-year continence rates post ARVUS and reported continence rates of 60.9%, 95.4%, 94.3% and 52.7%, 77.8%, 88.9% for the control group (*n* = 95) and ARVUS group (*n* = 36) respectively, at the 3- (*p* = 0.5269), 6- (*p* = 0.0077) and 12-month (*p* = 0.5100) stages [27]. Zorn et al*.*, trialled the efficacy of barbed polyglyconate suture versus standard monofilament for the posterior reconstruction and VUA, finding comparable continence rates in both groups (3 months *p* = 0.54, 6 months *p* = 0.67) (Table 2) [28]. In another RCT analysing ARVUS, Student et al*.* reported statistically significant improvements in urinary continence (0 pads/24 h) in the intervention arm at the 2-week (*p* = 0.005) and 1- (*p* ≤ 0.001), 6- (*p* = 0.013) and 12-month (*p* = 0.040) post-operative points [29]. The ARVUS group had continence rates of 62.5% (*n* = 20), 75% (*n* = 26) and 86.7% (*n* = 26) at the 1-, 6- and 12-month intervals [29]. In comparison, the control group reported rates of 14.7% (*n* = 5), 44.1% (*n* = 15) and 61.3% (*n* = 19) at respectively corresponding intervals [29]. The final study included in this review, by Sammon et al*.*, compared single- versus double-layer VUA. Two-year continence rates, defined via pad usage, were 80% for the control group (*n* = 57) and 82.6% for the double-layer VUA (*n* = 59) (*p* = 0.596) [30].

**Table 2:** Urinary continence rates in studies using different vesicourethral anastomosis techniques in RALP

First author	Study design	Validated questionnaire	Continence definition	Cases, n	Group	Urinary continence, %				Level of evidence [5]
						3 m	6 m	12 m	24 m	
**Choi’15 **[26]	RCT	EPIC	0 pads/24 h	79	BN-S	53.2	72.2	-	-	1b
				79	Control	59.9	74.7			
**Kovacik’19 **[27]	CT (part random)	ICIQ-SF	≤ 1 pad/24 h	36	ARVUS	52.7	77.8	88.9	-	2b
				95	Control	60.9	95.4	94.3		
**Zorn’12 **[28]	RCT	UCLA PCI	0 pads/24 h	33	PGS	81	92	-	-	1b
				33	Control	76	88			
**Student’16 **[29]	RCT	ICIQ-SF	0 pads/24 h	32	ARVUS	-	75	86.7	-	1b
				34	Control		44.1	61.3		
**Sammon’10 **[30]	RCT	None	0 pads/24 h	57	s-VUA	-	-	-	80	1b
				59	d-VUA				82.6	

*RCT* randomized controlled trial, *CT* (part random) partially randomised controlled trial, *EPIC* Expanded Prostate Cancer Index Composite, *ICIQ-SF* International Consultation on Incontinence Questionnaire -Short Form, *UCLA-PCI* University of California Los Angeles Prostate Cancer Index, *BN-S* bladder neck plication suture, *PGS* – barbed polyglyconate suture, *ARVUS* advanced reconstruction of vesicourethral support, *s-VUA* single-layer vesicourethral anastomosis, *d-VUA* double-layer vesicourethral anastomosis

## Discussion

Prostate cancer surgery aims to achieve maximum oncological clearance with the minimal possible impact on patient post-operative function and quality of life [2]. Since the introduction of robotic surgery, many different approaches have been developed for RALP. It is an exciting and novel area in urology, with advancements and refinements continuously being made to each technique. Unfortunately, there are few studies assessing and comparing these techniques in a rigorous scientific manner [3]. The application or omission of these methods is therefore, very often, down to surgeon preference. Unsatisfactory functional outcomes after prostatectomy impact patient quality of life, psychosocial behaviours and healthcare costs [27].

This review examines urinary continence outcomes of RCTs investigating RALP over the last 20 years. Post-RALP continence is influenced by preoperative patient characteristics, surgical techniques, follow-up intervals, surgeon experience and descriptive methodologies (i.e. continence definitions). Many papers have predictive factors for recovery of urinary continence post prostatectomy, with old age, less nerve-sparing and high BMI resulting in worse outcomes [2–4, 9, 14]. Nomograms predicting recovery of urinary continence post-RALP have been designed at 4- and 12-weeks [31] and 6-, 12- and 24-month [32] follow-up points. Pre-operative predictive measures of continence such as mean urethral length (MUL) have been described since 2002 when Coakley et al*.* noted a positive relationship between longer MUL and return to early continence [33]. A recent systematic review described a 200% increase in odds of returning to continence with a 10 mm increase in MUL [34].

Orgasm-induced urinary incontinence, or climacturia, is a common side effect post prostatectomy [35]. This symptom is gaining more attention due to the distress it causes to patients. Many mechanisms have been suggested to explain the pathophysiology, with none yet adequately tested. Currently, patients describe coping mechanisms such as voiding before intercourse and using condoms to capture urine incontinence after orgasm [36]. These strategies have been reported as ineffective [35]. Pelvic floor rehabilitation, penile loop mechanisms, artificial urinary sphincter, urethral sling surgery and the Mini-Jupette graft are suggested options [35]. Variable tension penile loops [37] and artificial urinary sphincter [38] were both associated with reduced climacturia in small case series.

There is no study or data to provide an agreement on the best surgical approach to treating prostate cancer. RALP is becoming the most popular and disseminating widely across the world, yet there is no definitive evidence to support this change in practice. Interestingly, Porpiglia et al*.*, noted a significant difference in favour of RALP over LRP regarding patient satisfaction post-operatively via the EPIC questionnaire [10]. They also reported a statistically significant improvement in urinary continence at 1- and 5-year post-RALP when compared to LRP [10]. Coughlin (RALP vs ORP) and Asimakapolous (RALP vs LRP) found no significant difference in continence at the 12-month period [9]. In the study by Giberti et al*.*, BT proved to have better short-term continence at 6 months, but RALP and BT were comparable with no difference at 12 months [12].

In terms of RALP techniques, Akand et al*.* investigated urinary continence outcomes whilst comparing the trans- and extra-peritoneal approaches to RALP. They found no meaningful difference in continence at the 6-month interval. Although they did not find a difference in continence rates between groups, they concluded E-RALP is an alternative to T-RALP due to the reduced rates of bowel injury, earlier return to diet and similar functional and oncological results [22]. In a unique study by Hyunh et al*.*, regional hypothermia was trialled as part of the RALP operation via an endorectal cooling balloon, but the technique failed to demonstrate any benefit in return urinary continence [23]. Delayed versus standard ligation of the dorsal venous complex was studied by Antonelli et al*.* in 2018. They concluded that both approaches have comparable functional outcomes, with some benefit to reduced risk of apical positive surgical margins in the delayed ligation arm of the study [24]. The addition of pre-operative pelvic floor muscle exercises to a post-operative rehabilitation regime did not show any benefit to return of continence in study by Geraerts et al*.* [25].

The use of autologous urethral slings was studied in four papers in this review. Nguyen found that sling placement was not associated with improved return to continence at 6 months [14]. Bahler et al. had similar conclusions, with no significant increase in continence attributed to the addition of a biologic bladder neck sling [15]. In two different studies by Cestari, sling vs control and two-arm vs six-arm slings were compared. In the first study, there was statistically significant improvements in continence (defined as zero pads per day) in the sling group at the 1-, 3-, 6- and 12-month periods [16]. In the latter study, they found statistically significant improvements in urinary continence within 30 days post-RALP but did not note a meaningful change at 1-year [17]. There was no significant increase to operative time in either Cestari trial [16, 17], while Bahler did report a significant increase associated with sling placement [15]. Interestingly, Nguyen et al*.* did not report operative times [14].

Atug et al*.* and Hurtes et al*.* investigated the impact of total anatomical reconstruction after RALP on return to continence. Atug proposed improved continence with reconstruction of the anterior association of the pubic symphysis to the DVC followed by posterior rhabdosphincter and Denonvilliers’ fascia restoration. They found statistically significant improvements in continence at 4 weeks post-RALP but noted overall similar rates of continence at 1 year [18]. Hurtes et al*.* applied a similar technique with improvements noted at 1 and 3 months post operatively but with no long-term difference between the groups [19]. In a 2011 study by Sutherland, posterior rhabdosphincter reconstruction was added to their regular RALP technique. The results showed no increase to recovery of early urinary continence with reconstruction of the posterior musculofascial plate [21]. Jeong et al., described a similar technique stating “We devised a 1-step posterior reconstruction technique that opposes the median dorsal raphe only to the posterior counterpart of the detrusor apron rather than to Denonvilliers’ fascia.” However, the results only showed a small benefit to early social continence between the groups [20].

In the subgroup analysing vesicourethral anastomosis techniques, Advanced Reconstruction of Vesicourethral Support (ARVUS) was examined by Student and Kovacic [27, 29]. Kovacic reported no difference between ARVUS and the control arm but noted a statistically significant association between nerve-sparing, unilateral or bilateral, and improved continence [27]. Student et al. described statistically significant improvements in continence in the ARVUS group at every stage from 2 weeks to 1 year post-RALP [29]. They confirmed this data using the International Consultation on Incontinence Questionnaire—Short From (ICIQ-SF), also finding statistically significant results via this definition of continence [29]. Investigation of a bladder-neck plication suture by Choi et al*.* highlighted inferior outcomes in the investigation arm compared to the control group, although these differences were not statistically significant [26]. Zorn et al. compared barbed 3–0 polyglyconate sutures to the control suture 3–0 poliglecaprone 25 monofilament for the VUA [28]. They discovered no difference in urinary continence rates between groups up to 6 months post-RALP; however, they did recommend the polyglyconate suture as a safe and cost-effective alternative, with decreased anastomosis time, cost reduction and decreased need to readjust suture tension [28]. In 2010, Sammon et al. compared a single versus double-layered suturing of the VUA. They stated no substantial difference in continence between the groups [30].

Menon investigated the Retzius-sparing RALP (RS-RALP) technique. The study reported improvements in very early (< 1 month) urinary continence post RS-RALP when compared to the traditional anterior approach. This advantage was not as prominent at long-term follow-up [13]. In a recent systematic review, authors demonstrated statistically significant improvement in urinary continence at 1, 3, 6 and 12 months post-RALP when the RS-RALP was applied [4].

**Fig. 4:**
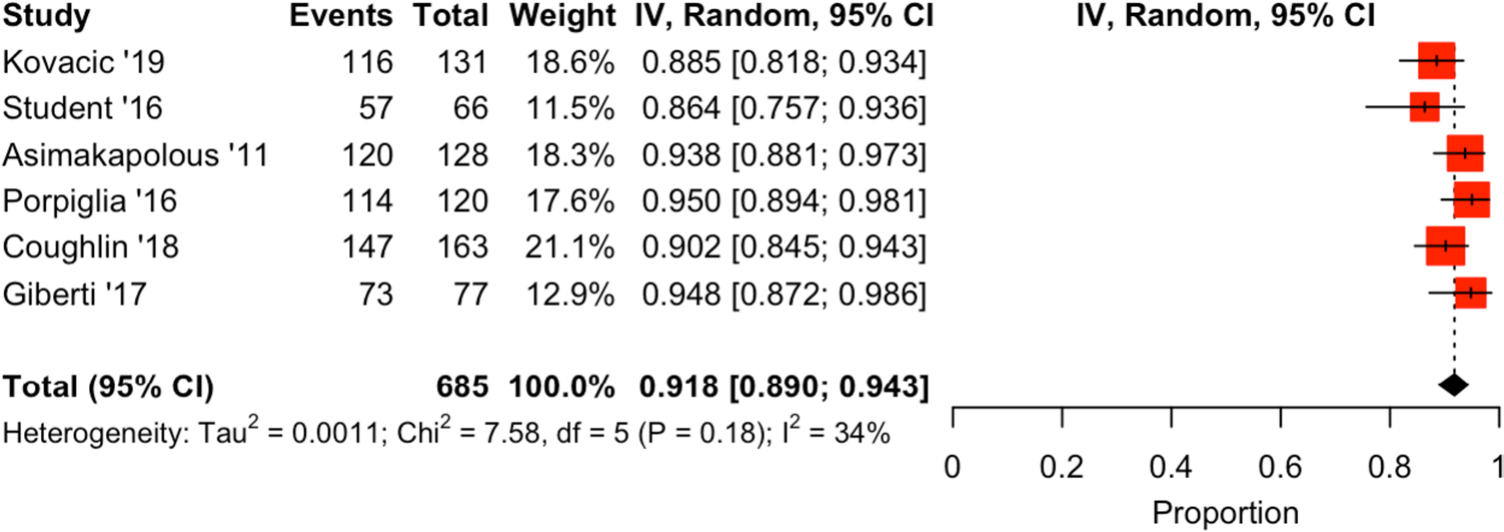
Cumulative analysis and forest plot—percentage of men continent post-RALP at 12 months

Interestingly, there were conflicting reports on multiple techniques included in this systematic review. In terms of sling placement, Cestari recorded significant improvements using a two-arm autologous sling, but failed to reproduce long-term results with the six-arm sling replacement [16, 17]. While Nyugen and Bahler noted no meaningful improvement with the addition of a sling [14, 15]. Atug et al*.* recorded improved early continence in their intervention group (anatomical reconstruction), whereas Hurtes did not reproduce these results with a similar practice [18, 19]. ARVUS was successfully employed by Student et al*.*, but Kovacic and team did not repeat this same success [27, 29]. When comparing approach to prostatectomy, RALP was shown to have significantly improved continence at all follow-up points when compared to LRP by Porpiglia et al*.* [10]; however, this was not replicated in a paper by Asimakapolous et al. [11]. BT was shown to have favourable early continence rates and comparable long-term continence when compared to RALP by Giberti et al. [12]. There was no significant difference noted when comparing post-ORP and post-RALP continence [9]. Pooled analysis of 12-month continence rates can be seen in Fig. 4.

The main limitation of this review is the significant heterogeneity between studies; thus, a formal meta-analysis was not performed. It is well described that RCTs examining surgical technique are difficult to compare for a number of reasons. These include inability to account for individual surgical skill, learning curves associated with changes in technique, different equipment and setup used in different centres (e.g. different robots, variance in port placement, experience of assistant). This review excluded comparative and observational studies; however, much of the literature on this topic is non-experimental data. There were no studies that met the inclusion criteria that investigated the perineal approach to RALP. This is a potential weakness, as this method is employed in certain practices.

## Conclusion

Continence rates following RALP were favourable compared to LRP, comparable to ORP and less favourable compared to BT. However, the literature suggests post-RALP continence can be further improved with alterations in robotic technique. Improved early urinary continence with RALP was noted in many studies, but the long-term benefit to continence was often comparable with control groups. Increasing age, higher BMI and lack of nerve-sparing were associated with worse continence rates.

## Abbreviations

ARVUS: Advanced reconstruction of vesicourethral support
AS + PR: Anterior suspension and posterior reconstruction
AUASS: American Urological Association Symptom Score
BT: Brachytherapy
DVC: Dorsal vascular complex
d-DVC: Delayed dorsal vascular complex ligation
s-DVC: Standard dorsal vascular complex ligation
EPIC: Expanded Prostate Cancer Index Composite
E-RALP: Extra-peritoneal robot-assisted laparoscopic radical prostatectomy
IPSS: International Prostate Symptom Score
ICIQ-SF: International Consultation on Incontinence Questionnaire -Short Form
LRP: Laparoscopic radical prostatectomy
MUL: Mean urethral length
n: Number of cases
ORP: Open Retropubic Radical Prostatectomy
PICO: Patient Intervention Comparison Outcome criteria
PFME: Pelvic floor muscle exercises
PRISMA: Preferred Reporting Items for Systematic Reviews and Meta-Analyses guidelines
PSA: Prostate-specific antigen
RALP: Robot-assisted laparoscopic radical prostatectomy
RCT: Randomised controlled trial
R-HT: Regional hypothermia
RS-RALP: Retzius-sparing robot-assisted laparoscopic radical prostatectomy
STARR: MSKCC (Memorial Sloan Kettering Cancer Centre) STAR (Symptom Tracking and Reporting)
T-RALP: Trans-peritoneal robot-assisted laparoscopic radical prostatectomy
TR: Total reconstruction
UCLA–PCI: University of California Los Angeles Prostate Cancer Index
VUA: Vesicourethral anastomosis
VAS: Visual analogue scale
